# The impact of unconditional cash and food assistance on contraceptive expenditure of rural households in Coastal Bangladesh: Evidence from fuzzy RDD

**DOI:** 10.1371/journal.pone.0262031

**Published:** 2022-01-21

**Authors:** MD. Abdul Bari, Ghulam Dastgir Khan, Bing He, Yuichiro Yoshida

**Affiliations:** 1 Graduate School for International Development and Cooperation, Hiroshima University, Kagamiyama, Higashi-Hiroshima, Japan; 2 School of Business, Jiangsu Ocean University, Haizhou District, Lianyungang, Jiangsu, China; 3 Graduate School of Humanities and Social Sciences, Hiroshima University, Kagamiyama, Higashi-Hiroshima, Japan; Shahjalal University of Science and Technology, BANGLADESH

## Abstract

This study investigates the impact of unconditional cash and food (UCF) assistance on the monthly contraceptive expenditure of rural households in coastal Bangladesh using a fuzzy regression discontinuity design (RDD). Eligibility for UCF assistance was based on the running variable of land ownership in acres. We used eligibility as an instrumental variable to estimate the local average treatment effect of UCF assistance on contraceptive expenditures. The results show that UCF assistance results in increases in monthly contraceptive expenditures.

## 1. Introduction

Bangladesh, one of the world’s most densely populated countries, has been using unconditional cash and food (UCF) assistance, a major type of social safety net (SSN), as a policy tool to provide resources to vulnerable households encouraging consumption, savings, and investment. UCF assistance has a significant impact on household expenditures [1] and increases the savings of poor households [2]. By increasing savings, UCF assistance can decrease the financial burdens of vulnerable households [3], allowing an increase in expenditures on items such as contraception.

UCF, although not intended to change childbearing decisions directly, can still have unintended positive or negative effects on reproductive outcomes [4]. A few studies have estimated the impact of cash or food assistance programs on procreation decision making as an unintended outcome [4–7]. The results are mixed. In addition, the findings differ depending on the context of different countries due to diverse culture, values, and preferences. Developed countries, such as the United States and Canada used cash transfer as a means to increase fertility, whereas developing countries in South Asia, Latin America and Africa used cash transfer as a means to reduce fertility [6]. In [5], the authors used a linear regression model to argue that cash transfers increase contraceptive use in India. In contrast, the authors in [6] applied difference in differences to claim that cash transfer programs have a positive impact on fertility in Argentina. The authors in [4] employed fuzzy regression discontinuity design (RDD) and instrumental variable (IV) estimation and argued that cash transfer programs did not have an impact on contraceptive use in Ecuador. However, the authors in [4] mentioned that their IV was weak. Cash transfer programs have a significant impact on fertility in Canada [8], but welfare policies for young mothers have no significant impact on fertility [9]. Empirical findings are not conclusive in identifying the impact of cash transfer on fertility [6,10]. Furthermore, cultural, religious, social values create differences in contraceptive choice and usage. Male and female relationship dynamics often determine the choice of contraceptive. For instance, in a male dominated society like Bangladesh, oral pills are the mostly used contraception while in women-empowered counties such as Canada sterilization (male and female) is widely used. Moreover, for Bangladesh, as a Muslim majority country sterilization is considered as a religiously prohibited practice. Consequently, the impact of cash or food assistance on contraceptive use remains a significant question to be analyzed rigorously [6]. Our study addresses this gap by examining whether UCF assistance has any causal impact on monthly contraceptive expenditure in the context of developing countries. This study addresses the research question as to whether cash or food assistance can be used as a tool to increase the usage of contraceptives. Furthermore, this study aims to understand the financial mechanism needed to increase the usage and quality of contraceptives.

Contraception expenditures have been considered as an indication of the increasing ability to invest in women and child health, willingness to use contraception, and childbearing decision making for vulnerable households [11]. This study further checks the mechanism of the family investment model propounded in [12] suggesting that vulnerable households invest in human capital by increasing expenditure on family planning when budget constraints are lifted. Increased expenditure on contraceptives can be associated with an increased investment in women and child health in vulnerable households.

Bangladesh, with a population density of around 1050 people per square kilometer, had a target of achieving a fertility rate of 1.7% in 2014; the actual fertility rate was 2.3% [13]. Increasing contraceptive use has become a key policy objective since initiating the first population control policy in 1976. According to the 2012 Population Policy, the target was to achieve a contraceptive use rate of 72% by 2015, but the actual rate was 60% [13]. This study aims to explore whether UCF assistance can be adopted as a policy tool to increase the prevalence of contraceptive use among vulnerable households. Usage of contraceptives has been promoted in Bangladesh since 1976 to control population growth and both hormonal and intrauterine contraceptives are available in drug stores or family health care centers in Bangladesh. Hormonal contraceptives, such as oral pills, are inexpensive in comparison to other long-term methods of contraception. While religious and traditional beliefs discourage the usage of permanent contraception methods such as sterilization surgery, they are still widely available. In Bangladesh, 27% of married couples use oral pills, 12.40% use injectable contraceptives, 6.4% use condoms, 4.6% use female sterilization, 1.2% use male sterilization, 1.7% use implants, and 0.6% use intrauterine devices [13]. According to traditional practices, the choice of contraceptive is often decided by the male partner due to male dominance and contraceptive expenditures are also often borne by them, in Bangladesh. Moreover, male partners are often indifferent to the use of contraceptives, but female partners are keener to use them to control reproduction and reduce unintended pregnancies. Thus women empowerment is expected to have a significant positive impact on contraceptive choice and usage [14]. UCF assistance in Bangladesh mainly aims at women empowerment [15] by ensuring women’s access to cash and food assistance. As most of the women in vulnerable households in Bangladesh are without any income and job, even a small amount of grant of BDT 400 provides the women with resources to spend according to their choice and need. Moreover, the amount of money required for the most commonly used contraception, oral pills, is around BDT 150 per month. Thus it remains a relevant question to examine whether UCF assistance can increase out of pocket expenditure on contraception. UCF assistance aims at ensuring economic independence for women in vulnerable households, and the economic dependence of women can be manifested by regulating fertility [16]. This study further analyzes the bottom-up approach of empowering vulnerable women through cash and food assistance to increase investment in woman and child health care represented by expenditure on contraception. This study considers rural households in coastal Bangladesh as the unit of analysis. The coastal zone of Bangladesh is one of the most densely populated [17] and most vulnerable coastal regions of the world [18]. Along with high poverty and inequality, rural coastal regions lack adequate health care delivery [18]. Examining whether UCF assistance can increase expenditure on health issues, such as contraception, is a relevant concern. We applied fuzzy RDD in which treatment was partially determined based on the running variable being just below or above a threshold. Land ownership was the running variable, which partially determined UCF assistance when a household owned land just below or equal to half an acre; households that own land equal to or below 0.5 acre received priority UCF assistance [19]. Fuzzy RDD validity was determined using the manipulation test proposed in [20].

The remainder of this paper is structured as follows: Section 2 discusses the policy background of UCF assistance. Section 3 provides an overview of the data used in this study and the identification strategy, followed by the findings in Section 4. Section 4 presents the discussion and conclusions.

## 2. UCF assistance in Bangladesh

Since independence in 1971, a number of SSN UCF assistance programs in Bangladesh have had the goal of reducing poverty and inequality. Initially, the cash and food assistance efforts were limited to food rations and post-disaster relief provided to vulnerable districts. Since 1997, cash and food assistance programs expanded to the household level with the aim to addressing the demographic and social challenges faced by the poorest households. Most of the UCF assistant programs aim at woman’s empowerment through ensuring women’s access to cash assistance. However, the effectiveness of UCF assistance remains a question because of the small grant amounts.

A national SSN program policy was formulated in 2015 to regulate and implement SSN programs. In fiscal year (FY) 2016, total Bangladeshi Taka (BDT) 245.21 billion was allocated as cash transfers, conditional cash transfers, and food assistance. The budget for SSN programs amounted to 13.1% of the annual budget and 2.08% of the GDP for FY2016 [21]. The Old Age Allowance Program and Widow, Destitute, and Deserted Women Allowance are two major UCF programs in Bangladesh. The very first formal SSN program, the Old Age Allowance Program, was introduced in FY1998 to ensure the social and economic security of the poor and vulnerable elderly persons. In FY2016, a total of BDT 14.40 billion was allocated under this program and around 3 million elderly people were given BDT 400 on a monthly basis [21]. The second formal UCF program of Bangladesh, Widow, Destitute, and Deserted Women Allowance was introduced in FY 2000. In FY 2016, a total of BDT 5.34 billion was allocated under this program and the estimated number of beneficiaries receiving BDT 400 monthly under this program was 1.10 million [21]. The Vulnerable Group Feeding (VGF), Vulnerable Group Development (VGD), Gratuitous Relief (GR), and Test Relief programs are food assistance programs implemented to reduce food insecurity in vulnerable households. A total of BDT 14.61 billion was allocated for the VGF program in FY 2016. Moreover, BDT 9.90 billion was allocated under VGD program in FY 2016 and around 177,000 metric tons of food grains were distributed among the beneficiaries [21]. The GR program allocated 27,000 metric tons of rice, with approximately 1.3 million households benefiting in FY 2016. The Ministry of Disaster Management and Relief administers the Test Relief program for disaster-affected households. Approximately 0.20 million metric tons of rice and wheat were allocated under this program in FY 2016 [21].

## 3. Materials & methods

### 3.1 Description of the data

This study included 8193 rural coastal households as the unit of analysis. The observations of this study come from the Household Income and Expenditure Survey (HIES) 2016–2017, a national survey conducted at five-year intervals [22]. This was a cross-sectional dataset with 46,076 observations. The households included in this study were from 19 coastal districts of Bangladesh. The coastal districts are Bagerhat, Barguna, Barisal, Bhola, Chandpur, Chittagong, Cox’s Bazar, Feni, Gopalgonj, Jashore, Jhalokathi, Khulna, Lakshmipur, Narail, Noakhali, Patuakhali, Pirojpur, Satkhira, and Shariatpur. The sample was restricted to rural households in coastal districts, which reduces the sample size from 46076 to 9520 households. To include only vulnerable households, the households that own financial assets of more than BDT 50,000.00 (USD 1 = BDT 86.09 approximately) were excluded, reducing the sample size to 8700. The dataset does not have any proper data on income of the households so only the amount of financial asset has been used to define vulnerable households. Moreover, households that received UCF assistance before 2015 were excluded, reducing the sample size to 8559. In addition, households that had sold or purchased land in the last 12 months were excluded, reducing the sample size to 8220. In addition, observations with missing running variables were excluded, and the final sample size was 8193 households.

The HIES 2016–2017 contains a section on SSN programs. This study considers the households that received the last payment from UCF assistance programs in 2015 or 2016 as included and households were not included if otherwise. The HIES 2016–2017 contains a section about land ownership from which we generated the running variable. Total cultivable land was treated as land ownership. For estimation ease, land ownership was normalized with a new cutoff of 0 by subtracting land ownership from the cutoff value of 0.5 acres. Contraceptive expenditure data was generated from the section on health expenditure information in HIES 2016–17.

### 3.2 Identification strategy

Fuzzy RDD was used to estimate the impact of UCF assistance on monthly contraceptive expenditures. The outcome variable is monthly contraceptive expenditure and UCF assistance is the endogenous variable in our study. Fuzzy RDD has been applied in the study to estimate treatment effect of UCF assistance because it effectively removed endogeneity problem by local randomization just below and just above the cutoff of a running variable. To address the selection bias and to isolate the causal impact, we used eligibility criterion for UCF assistance based on land ownership equal to or less than 0.5 acre as an IV in this study. The eligibility dummy, which is used as an IV, takes a value of 1 if a household owns land below or equal to half an acre and it takes a value of 0 if otherwise. In other words, the jump in the treatment status around the cutoff, 0.5 acres of land ownership is used as the IV. One of the striking features of fuzzy RDD is that the conditions required to be an IV can be tested more easily than in a conventional IV estimation. Firstly, the relevance condition of the IV being correlated to the endogenous variable can be checked in the first stage estimation and secondly, the exclusion restriction condition of the IV being uncorrelated to the outcome variable can be tested by checking the continuity of the running variable and the continuity of other covariates around the cutoff of the running variable. Checking continuity in the running variable and continuity in the pretreatment covariates can be also interpreted as tests of the exogeneity condition which requires that the IV is randomly assigned. The absence of any significant jump around the cutoff would suggest that all the relevant covariates are on average same around the cutoff and that the IV is not likely to have any direct impact on the outcome variable.

A household is eligible to receive UCF assistance if it owns land below or equal to the cutoff of 0.5 acre. In this study, there are three types of households: compliers, always takers, and never takers. Compliers are households that receive safety net cash or food because of eligibility based on the land ownership threshold of 0.5 acre and would not receive it if not eligible. Households are always takers if they receive UCF assistance, despite not being eligible. Households are never takers if they do not receive UCF assistance despite being eligible.

In fuzzy RDD, the jump in the treatment probability at the cutoff is smaller than one; therefore, it involves IV type estimation. This estimation measures the local average treatment effect for the compliers, instead of the overall average treatment effect. In this study, treatment receipt was estimated using the first-stage estimation equation as follows:

$$X_{i}=\alpha_{0}+\alpha_{1}R_{i}+\pi E_{i}+u_{i}$$

Here, *X*_*i*_ denotes a dummy variable for treatment status, which takes the value of 1 if a household has received any UCF assistance in 2015 or 2016 and it takes value of 0 otherwise. *E*_*i*_ is the eligibility dummy and acts as the IV and takes the value of 1 when *R*_*i*_ is less than or equal to the eligibility cutoff: *E*_*i*_ = 1 (R_i_≤0.5). *R*_*i*_ is the running variable, and land ownership in acres. *π* equals the jump in the probability of the treatment status around the cutoff.

From the first-stage estimation equation, the estimated ${\hat{X}}_{i}$ is found and the estimated ${\hat{X}}_{i}$ is used as an independent variable in the second-stage estimation equation, which takes the form

$$Y_{i}=\beta_{0}+\beta_{1}R_{i}+\eta_{c}{\hat{X}}_{i}+\varepsilon_{i}$$

where *Y*_*i*_ denotes the outcome variable. The local average treatment effect is denoted by *η*_*c*_. In other words, this study uses two-stage least squares estimation to obtain the local average treatment effect. The ratio of the change in the outcome variable and the change in treatment probability is the local average treatment effect in the fuzzy RDD setting. The choice of bandwidth around the threshold is a key consideration in RDD settings. We employ a CE-optimal bandwidth choice designed to minimize the coverage error of the interval estimator [23]. Moreover, we employed the local randomization-based RD estimate proposed in [23] as a robustness check.

## 4. Results

### 4.1 Summary statistics

Table 1 presents the summary statistics for all the data used in the analysis. The eligibility is 1 if land ownership is below or equal to 0.5 acre and 0 if otherwise. The table shows 84% households were eligible to receive UCF assistance and 16% households were not eligible. Moreover, 21% of the eligible households received UCF assistance and 15% of ineligible households received UCF assistance.

**Table 1:** Summary statistics of variables in this study.

Variables	Eligible Households	Noneligible Households
**Running Variable**		
Land Ownership (Acre)	0.052 (0.12)	2.27 (13.84)
**Treatment Variable**		
UCF Assistance	0.21 (0.41)	0.15 (0.36)
**Outcome Variable**		
Contraceptive Expenditure	17.53 (52.41)	16.06 (43.00)
**Covariates**		
Age of Head	44.50 (14.00)	50.16 (14.64)
Average Age	28.89 (12.60)	33.73 (13.41)
Gender (= 1 if Male Headed)	0.91 (0.28)	0.85 (0.36)
HH Members (< = 15 Years)	1.49 (1.14)	1.23 (1.09)
HH Members (Between 16 to 40 Years)	1.64 (0.92)	1.60 (1.04)
HH Members (Between 40 to 60 Years)	1.01 (0.77)	0.78 (0.75)
HH Members (>60 Years)	0.34 (0.58)	0.54 (0.71)
**Observation Number**	6917	1276

*Note*: Standard deviation is shown in the parentheses.

### 4.2 Main results

**Fig 1:**
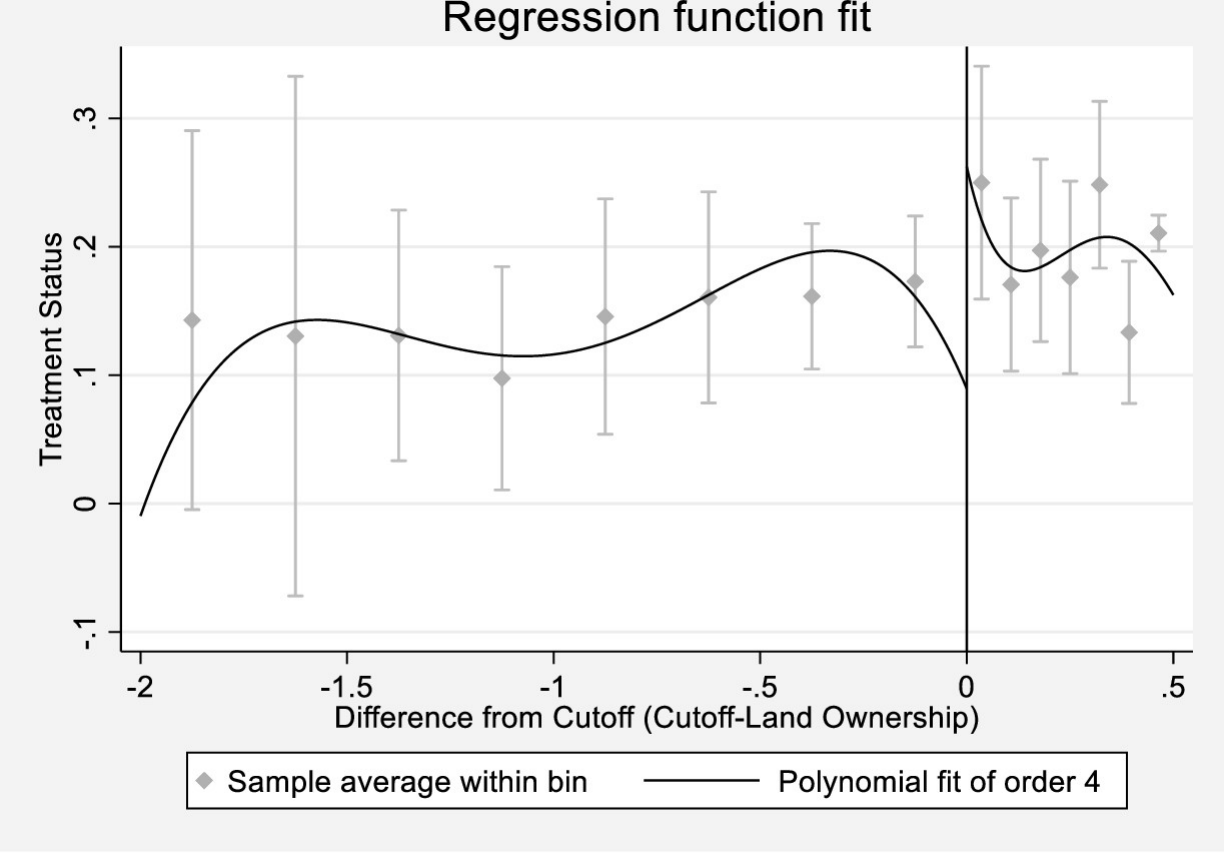
Discontinuity in treatment status around the cutoff. *Notes*: The horizontal axis is the difference from the cutoff in acres, and the vertical axis shows the treatment dummy. Every dot in the graph represents the sample average within bin, whereas every line represents the fourth order polynomial approximation of the outcome variable. The vertical line in the figure indicates 0 acres from the cut-off.

Table 2 presents the results from the first stage of the two-stage least squares regression. Robust RD estimates were reported. The result of first-stage estimation suggests that the eligibility dummy has a significantly positive impact on UCF assistance at 1% significance level. The first-stage estimation suggests that the eligibility dummy satisfies the relevance condition of IV estimation. We can conclude that the first stage is strong enough for second-stage estimation. The discontinuity in the treatment variable at the cutoff has been demonstrated in Fig 1, which shows significant jump at the cutoff of the running variable, suggesting discontinuity of treatment probability at the cutoff.

**Table 2:** First-Stage estimation of the effect of eligibility dummy on UCF assistance.

**Independent Variable**	**Dependent Variable** UCF Assistance Receipt (1 if household received UCF assistance)
Eligibility Dummy (1 if household owns land equal to or less than 0.5 acres)	0.24*** (0.08)

*Note*: Standard errors are in parentheses. Significance levels use a robust method where * for p < 0.10, ** for p < 0.05, and

*** for p < 0.01. “Robust” estimates use bias-corrected coefficient estimators and robust variance estimators.

**Fig 2:**
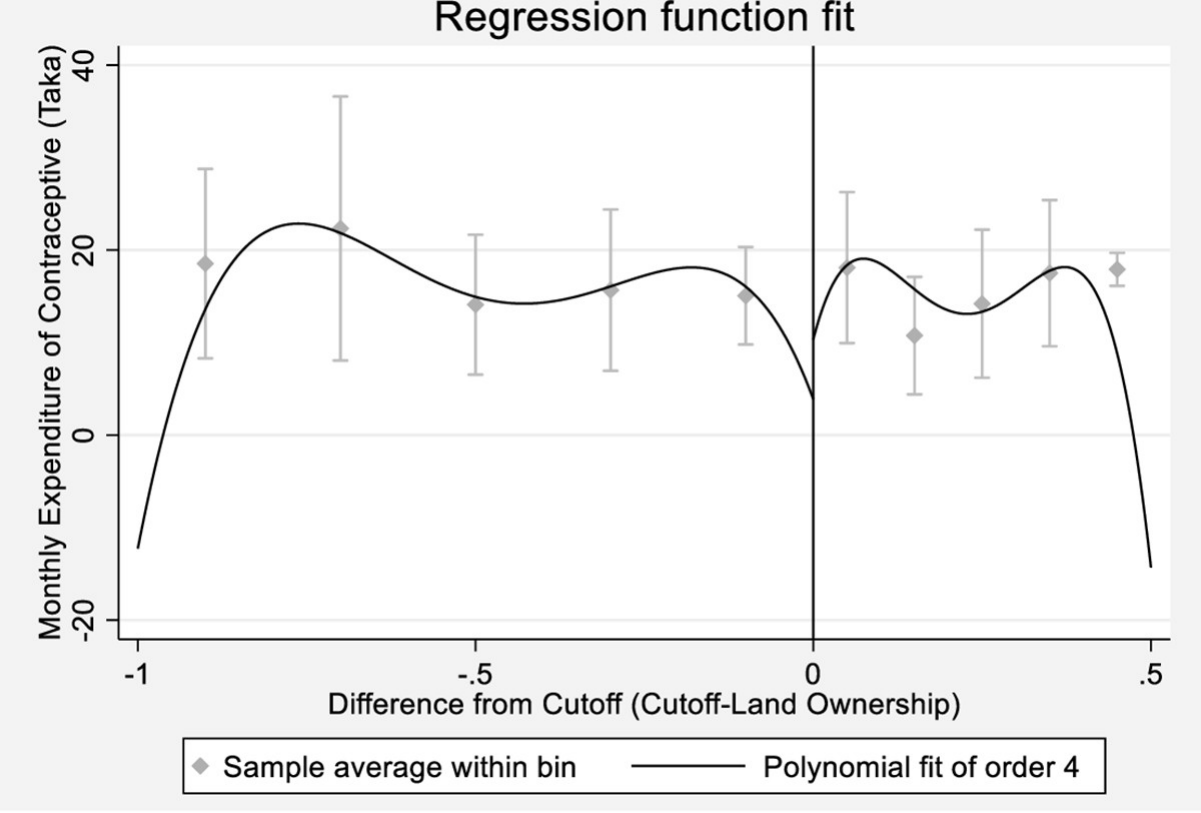
Discontinuity in monthly contraceptive expenditure around the cutoff. *Notes*: The horizontal axis is the difference from the cutoff in acres, and the vertical axis shows monthly contraceptive expenditure. Every dot in the graph represents the sample average within bin, whereas every line represents the fourth order polynomial approximation of the outcome variable. The vertical line in the figure indicates 0 acres from the cutoff.

Table 3 presents the local average treatment effect of UCF assistance on monthly contraceptive expenditure in BDT. Local linear regression discontinuity estimate shows that treatment has both positive and statistically significant impacts on monthly contraceptive expenditures at a 5% significance level. The second stage suggests that UCF assistance receipt increases monthly contraceptive expenditures of the compliers. Fig 2 shows the discontinuity in the monthly contraceptive expenditure with respect to the cutoff value of land ownership. The vertical line at 0 acres indicates that monthly contraceptive expenditure rises immediately beyond the cutoff, which is the measured local treatment effect shown in Table 3.

**Table 3:** Effect of UCF assistance on monthly contraceptive expenditure.

	**Outcome Variable** Monthly Contraceptive Expenditure in BDT	
**Treatment Variable**	Conventional Estimate	Robust Estimate
UCF Assistance Receipt (1 if household received UCF assistance)	74.82** (34.89)	72.31** (35.95)

*Note*: Standard errors are in parentheses. Significance levels use a robust method where * for p < 0.10

** for p < 0.05, and *** for p < 0.01. “Robust” estimates use bias-corrected coefficient estimators and robust variance estimators.

To identify the factors associated with contraceptive expenditure of the treated households; we conducted ordinary least squares (OLS) regression analysis. The results are reported in Table 4. The results show that only the average age of household members is statistically significant. Average age of the household members is negatively associated with contraceptive expenditure. The factors such as the number of children, literacy status, religion and childlessness do not have a statistically significant association with contraceptive expenditure. In addition, food assistance or cash assistance do not have any association with contraceptive expenditure. Moreover, the grant amount also does not have any association with contraceptive expenditure.

**Table 4:** Factors associated with contraceptive expenditure.

Dependent Variable Contraceptive Expenditure	Model: OLS
Factors	
Average Age of Household Members	-0.47*** (0.07)
Number of Children	0.97 (0.65)
Literacy Dummy (1 if HH Head is literate)	1.38 (1.65)
Religion Dummy (1 if Religion is Islam)	2.68 (11.35)
Childless Dummy (1 if HH has no child)	-0.58 (2.09)
Food Assistance (1 if HH received Food assistance)	-2.21 (1.80)
Grant Amount	-0.01 (0.01)

*Note*: Standard errors are in parentheses. Significance levels use a robust method where * for p < 0.10, ** for p < 0.05, and

*** for p < 0.01.

Our sample included every type of households and we depended on our identification strategy fuzzy regression discontinuity design to estimate the causal impact of the UCF assistance on contraceptive only around the cutoff of land ownership of 0.5 acre. The validity of Fuzzy RDD depends on the assumptions that all covariates are continuous around the cutoff. In other words, all the covariates need to be on average, the same around the cutoff. These issues are checked in the specification testing section.

### 4.3 Specification testing

Four major tests of the RDD setting were conducted to verify the validity of the design used in this study:

- Checking continuity in running variable
- Checking continuity in the pretreatment covariates
- Checking continuity of outcome variable at nondiscontinuity points
- Robustness check with a local randomization-based RD estimate

#### A. Checking continuity in the running variable

**Fig 3:**
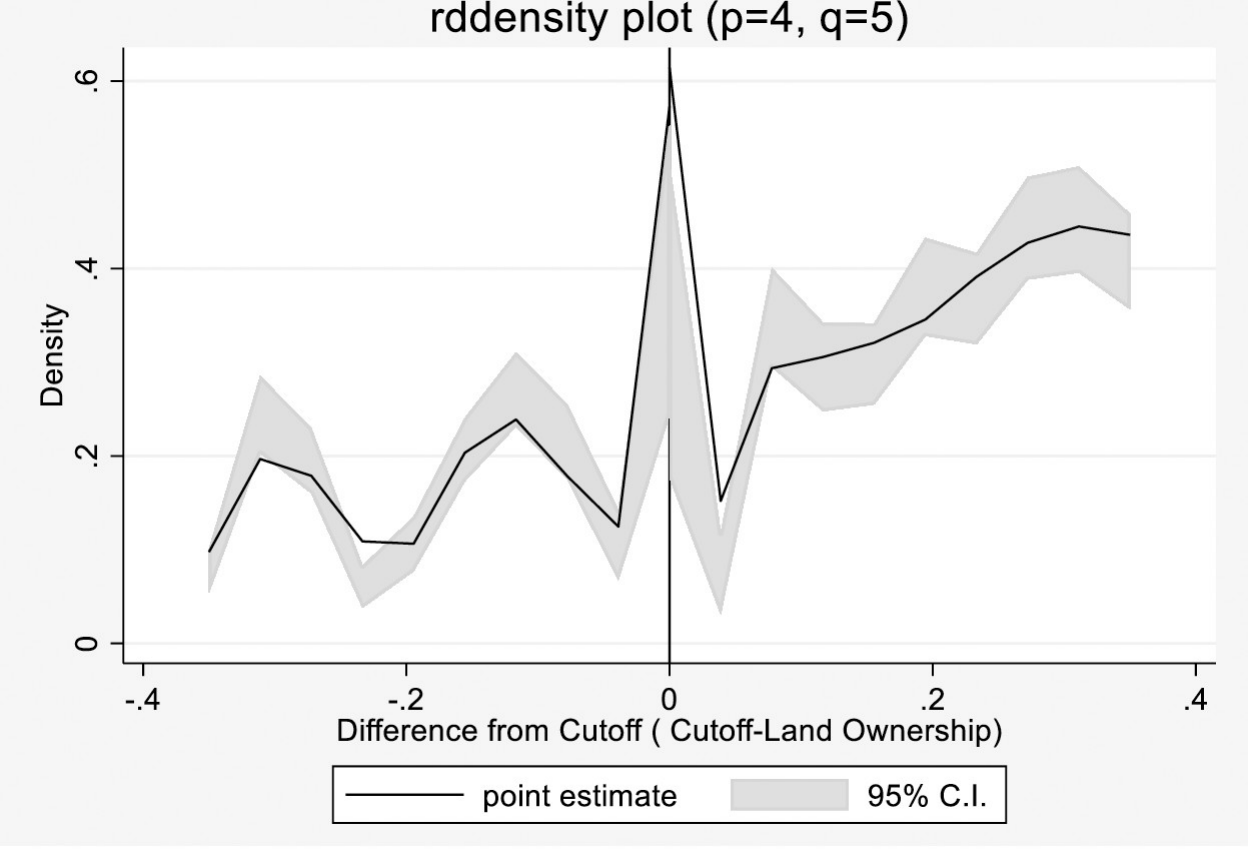
Checking discontinuity in the running variable.

RDD can be invalidated if significant discontinuity or manipulation occurs in the running variable threshold. To justify the use of fuzzy RDD, we tested the discontinuities in the conditional density of the running variable land ownership using a formal manipulation test based on local polynomial density estimators proposed by [24]. The density of land ownership should be continuous near this cutoff value to prove the claim that there is no systematic manipulation near the cutoff. The test determines whether a discontinuity exists in the density of the running variable at the known cutoff. The Cattaneo test confirms the absence of manipulation of the running variable land ownership. The graph is shown in Fig 3.

#### B. Checking continuity in pretreatment covariates

The validity of the RDD design requires continuity in pretreatment covariates. Therefore, we must first check whether there was any discontinuity in the covariates around the cutoff. Table 5 shows that the covariates’ RD estimates were not statistically significant, suggesting that the covariates were balanced around the cutoff. Thus, it can be concluded that there is no discontinuity in the covariates around the cutoff.

**Table 5:** RD Estimates to check discontinuity in covariates.

Covariates	RD Estimate
Age of HH Head	-0.01 (2.50)
Average Age	-0.64 (2.65)
Gender (= 1 if Male Headed)	-0.02 (0.05)
HH Members (< = 15 Years)	0.05 (0.17)
HH Members (Between 16 to 40 Years)	0.20 (0.15)
HH Members (Between 40 to 60 Years)	0.06 (0.15)
HH Members (>60 Years)	-0.01 (0.12)

#### C. Checking continuity of the outcome variable at nondiscontinuity points

To check the continuity of the outcome variable at nondiscontinuity points, we conducted RD estimates with different placebo cutoffs. The results are reported in Table 6, which shows that RD estimates with other placebo cutoffs are not statistically significant, confirming that there is no discontinuity in the outcome at nondiscontinuity points.

**Table 6:** RD estimates with placebo cutoffs.

Land Ownership	Monthly Expenditure on Contraceptive
Cutoff	RD Estimate
0.40 Acre	-959.97 (3782.20)
0.30 Acre	183.66 (723.61)
0.20 Acre	41.84 (138.78)

*Note*: Robust standard errors are shown in parentheses. Significance levels used a robust method where * for p < 0.10, ** for p < 0.05, and *** for p < 0.01.

#### D. Robustness check with a local randomization based RD estimate

Our main result comes from a continuity-based RD estimate, and we use a local randomization-based RD estimate as a robustness check, shown in Table 7. Under local randomization-based RD estimate, we assume that observations are randomly assigned to treatment in the short window of a finite sample [24]. The results show that UCF assistance reception increases contraceptive expenditure, which is consistent with our main result.

**Table 7:** Local Randomization-based RD estimate on the effect of UCF assistance.

Outcomes	Local Randomization based RD Estimate
Monthly Contraceptive Expenditure	46.49***

*Note*: Robust standard errors are shown in parentheses. Significance levels used a robust method where * for p < 0.10, ** for p < 0.05, and

*** for p < 0.01.

## 5. Discussion and conclusions

Vulnerable households are likely to have few resources to expend after meeting their basic needs like food, clothing, and accommodation. As a result, such households suffer from budget constraints preventing investment in health [12], and UCF assistance can ease the budget constraint allowing investment in health and reproduction. We found that UCF assistance has a positive effect on monthly contraceptive expenditure. The expenditure on contraception was used as an outcome variable in this study, not only as an indicator of vulnerable households’ willingness to invest in health matters, but also as an indicator of contraception use. The outcome can also be regarded as the reproduction decision of a household after receiving assistance in the form of cash and food. The policy implication is that UCF assistance can be used to increase expenditure on health matters such as contraception among vulnerable households in rural coastal districts. Furthermore, this study aims at finding the financial mechanism needed to increase usage and quality of contraceptive. One of the main reasons UCF assistance has a positive impact on the purchase of contraceptives is due to it being regarded as a way to empower women in vulnerable households [14], and woman empowerment has a positive impact on contraceptive expenditure. An increase in the contraceptive expenditure indicates that UCF assistance does empower women to invest in their health. Furthermore, contraception expenditure often constitutes a minimal amount of money in comparison to expenditure on other health issues but its implication on woman and child health is significant [25] as it reduces the possibility of unplanned pregnancies and complications for both mother and child.

Unconditional food or cash assistance allows vulnerable households to invest in human capital by increasing expenditure on contraception to control reproduction [4]. Expenditure on contraception implies not only an increase in the usage of contraception but also an increase in the quality of contraception choice [5]. High quality contraception means less side effects, and a marginal increase in expenditure on contraception results in a healthy life for sexually active women [26]. Besides, this study explored that food and cash assistance has no significant difference with relation to contraceptive expenditure. Furthermore, the grant amount also has no association with contraceptive expenditure. The literacy status, number of children, childlessness, and religious beliefs were not found to be associated with contraception expenses of vulnerable households. However, association does not imply causation in the present study. This study’s limitations need to be considered when interpreting the findings. First, the HIES data used in this study were cross-sectional; as a result, the time gap between treatment receipt and the outcome could not be measured properly. Second, the households were limited to those in the rural coastal districts of Bangladesh. Hence, this study has limited external validity. Third, a number of UCF assistance programs were considered as a whole, not separately.

However, to the best of our knowledge, this is the first study to estimate the causal impact of UCF assistance on contraceptive expenditure among rural coastal households in Bangladesh. This study suggests that family planning policymakers should consider UCF assistance as a way to motivate contraceptive use.
